# DJ-1 upregulates the Nrf2/GPX4 signal pathway to inhibit trophoblast ferroptosis in the pathogenesis of preeclampsia

**DOI:** 10.1038/s41598-022-07065-y

**Published:** 2022-02-21

**Authors:** Tingting Liao, Xia Xu, Xu Ye, Jianying Yan

**Affiliations:** Department of Obstetrics and Gynecology, Fujian Maternity and Child Health Hospital, Affiliated Hospital of Fujian Medical University, No. 18, Daoshan road, Gulou district, Fuzhou, 350001 Fujian Province China

**Keywords:** Cell death, Pathogenesis

## Abstract

Ferroptosis is a newly discovered mode of cell death that involves disorders in iron metabolism and the accumulation of reactive oxygen species (ROS) in the plasma membrane. Preeclampsia (PE) is a gestational idiopathic disease that is characterized by hypertension and albuminuria, begins after 20 weeks of pregnancy. DJ-1 is a prerequisite for activating and stabilizing Nrf2 to allow translocation to the nucleus to carry out further functions. Detecting the expression levels of DJ-1, the Nrf2/GPX4 signaling pathway and ferroptosis markers in placental tissues of pregnant women with and without PE. Analyzing the effects of the ferroptosis inducer (RSL3) and the inhibitor (Fer-1) on the mortality rate of BeWo cells and DJ-1+/+, DJ-1−/− BeWo cells. Ferroptosis markers (MDA concentration and morphology of trophoblast cells) and DJ-1 and its downstream the Nrf2/GPX4 signaling pathway increased significantly in PE pathological state. The expression levels of DJ-1 protein in the control group and the PE group were positively correlated with the expression levels of Nrf2/GPX4 signaling pathway protein, and negatively correlated with the MDA concentration. BeWo cells were sensitive to the ferroptosis inducer (RSL3) and the inhibitor (Fer-1). The high expression levels of DJ-1 in BeWo cells can resist ferroptosis by regulating the Nrf2/GPX4 signaling pathway. Ferroptosis is involved in the pathogenesis of PE. DJ-1 can mediate the trophoblast cells ferroptosis and play a protective role in the pathogenesis of preeclampsia by regulating the Nrf2/GPX4 signaling pathway.

## Introduction

Preeclampsia (PE) is a gestational idiopathic disease that is characterized by hypertension and albuminuria, begins after 20 weeks of pregnancy, and can cause damage in the liver, kidney, brain, and the functionality of other organs. The global incidence of PE is 5% to 8%. PE is one of the most common causes of maternal and perinatal morbidity and death, especially in developing countries^1^. Ferroptosis is a newly discovered mode of cell death that involves disorders in iron metabolism and the accumulation of reactive oxygen species (ROS) in the plasma membrane^2^. Ferroptosis can be triggered by excessive levels of Fe^2+^ or reagents that can increase the levels of intracellular ROS (such as NADPH oxidase)^3^. Ferroptosis can be pharmacologically inhibited by iron chelating agents (e.g., deferrioxamine), lipophilic antioxidants (e.g., ferristatin), and enzymes that can reduce lipid peroxidation and inhibit iron uptake^4^. However, ferroptosis does not respond to inhibitors that are related to apoptosis or necrosis^5^. Over recent years, there has been significant interest in the correlation between increasing ferroptosis and lipid peroxidation in plasma membrane and the pathogenesis of PE^6^. Many studies have observed the characteristics of ferroptosis in the physiological and pathological states of PE^7^. In a previous study, Siddiqui et al.^8^ reported that the concentration of malondialdehyde (MDA) in the serum of pregnant women with PE and eclampsia was significantly increased. In another study, Yang et al.^9^ reported that the accumulation of lipid peroxidation can inhibit a variety of cellular functions in trophoblast cells, including invasion, and participate in the pathogenesis of PE. These findings suggest that cellular ferroptosis is involved in the pathogenesis of PE, although the specific mechanisms underlying these observations remain unclear.

DJ-1 is a small molecular binding protein that is located in cell membranes. DJ-1 is an important sensor for the redox state in cells and a prerequisite for activating and stabilizing Nrf2 to allow translocation to the nucleus to carry out further functions^10^. Usually Nrf2 binds to Kelch-1-like epichlorohydrin-associated protein-1 (Keap-1) in the cytoplasm in an inhibitory state. When stimulated by signals of extracellular oxidative stress, DJ-1 can dissociate Nrf2 and Keap-1 and Nrf2 can translocate into the nucleus to bind with antioxidant response element (ARE)^11^ and induce high expression levels of a number of downstream antioxidant enzymes, including glutathione peroxidase 4 (GPX4) and superoxide dismutase (SOD), to protect the body from toxic substances^12^. In a previous study, Cao et al.^13^ reported the integration of DJ-1 in ferroptosis; specifically, these authors identified that the inhibition of DJ-1, both in vivo and in vitro, can effectively enhance the sensitivity of tumor cells to inducers of ferroptosis and that the depletion of DJ-1 plays a role in promoting ferroptosis by destroying the formation of S-adenosine homocysteine hydrolase tetramer and reducing its activity. In 2013, Kwon et al.^14^ reported that the expression levels of DJ-1 mRNA in the placentas of a PE group were significantly higher than those in a control group. It has been speculated that the overexpression of DJ-1 protein in the placenta of patients with severe PE act as a compensatory mechanism for hypoxia, although the specific mechanisms involved are not clear.

Therefore, we detected and analyzed the expression levels and correlations of DJ-1, the Nrf2/GPX4 signaling pathway, and markers of ferroptosis in the placental tissues of pregnant women with and without PE in the present study. We also investigated the effects of a ferroptosis inducer (RSL3) and inhibitor (Fer-1) on the mortality of DJ-1+/+ and DJ-10−/− BeWo cells. Our overall aim was to determine whether DJ-1 inhibits ferroptosis in trophoblast cells and protects in the pathogenesis of PE by regulating the Nrf2/GPX4 signal pathway.

## Materials and methods

### Patient recruitment and sample acquisition

We recruited 60 pregnant women who delivered in the Department of Obstetrics of Fujian Maternal and Child Health Hospital between November 2020 and March 2021. We divided these patients into two groups (30 cases per group): (1) PE group: the pregnant women with PE, and (2) a control group in which women underwent cesarean section due to a history of cesarean section, abnormal birth canal, abnormal fetal position, or social factors but without pregnancy complications. The diagnostic criteria of PE were in accordance with the ninth edition of Obstetrics and Gynecology (People’s Health Publishing House). All pregnant women in the PE group were delivered by cesarean section because of surgical indications. The pregnant women in the two groups were not in labor and the fetal membranes were not ruptured during cesarean section. Patients with other pregnancy complications or medical/surgical complications were excluded from the study. All pregnant women provided informed consent and signed an informed consent form. The experimental procedure complied with the moral standards of the local and national human experimental committees and the Declaration of Helsinki. The Ethics Committee of Fujian Maternity and Child Health Hospital approved all protocols (Reference: 2021KLRD631).

Human placental tissue was collected during cesarean section. Once the placenta had been delivered, we immediately removed a portion of placental villous tissue (1.0 cm^3^) from the root of the umbilical cord. We ensured that this procedure was carried out under aseptic conditions and made sure that tissue was only removed from areas on the placenta that did not show bleeding, infarction, or calcification. Tissue samples were rinsed with cold normal saline and placed in 5 ml cryopreservation tubes, frozen immediately in liquid nitrogen, and then transferred to a − 80 °C medical cryopreservation refrigerator (Thermo Scientific) for preservation.

### Determination of malondialdehyde (MDA) concentration

The MDA reaction system (Nanjing Jiancheng Biological Engineering Research Institute) was prepared in accordance with Table 1. Samples were heated at 95 °C for 40 min in a water bath and then cooled with running water. Then, samples were centrifuged at 3500–4000 rpm for 10 min. The supernatant was removed and measured on a microplate spectrophotometer at 532 nm. MDA content in tissue (nmol/mgprot) = (OD_test_ − OD_control_)/(OD_standard_ − OD_blank_) × standard concentration (10 nmol/ml) ÷ protein concentration of the sample to be tested (mgprot/ml).

**Table1:** The MDA reaction system.

	Standard tube	Blank tube	Test tube	Control tube
10 nmol/ml standard substance (ml)	a			
Absolute ethanol (ml)		a		
Test sample (ml)			a	a
Reagent1 (ml)	a	a	a	a
Mixing				
Reagent2 (ml)	3	3	3	3
Reagent3 (ml)	1	1	1	
50% glacial acetic acid (ml)				1

### Observation of cell morphology

Placental tissues were embedded in electron microscope fixing solution (Servicebio, Wuhan, China) for 2–4 h. The tissue was rinsed in 0.1 m phosphate buffer PB (PH7.4) (Pharmaceutical Group Chemical Reagent Co., LTD) for 3 times (each time for 15 min) and fixed at room temperature (20 °C) for 2 h in 1% Osmic acid (Pharmaceutical Group Chemical Reagent Co., LTD) · 0.1 m phosphate buffer PB (PH7.4). Rinse with 0.1 m phosphate buffer PB (PH7.4) for 3 times again, each time for 15 min. The tissue was successively put into 50–70–80–90–95–100% alcohol (Pharmaceutical Group Chemical Reagent Co., LTD)-100% acetone (Pharmaceutical Group Chemical Reagent Co., LTD) for dehydration, each time for 15 min. Then, the tissue was permeated for 2–4 h in acetone: 812 embedding agent (SPI, 90529-77-4) = 1:1, overnight in acetone: 812 embedding agent = 1:2, and in pure 812 embedding agent for 5–8 h. The pure 812 embedding agent was poured into the embedding plate, and the tissue samples were inserted into the embedding plate and then baked at 37 °C overnight. Put tissue in the oven at 60 °C and polymerize for 48 h and section at 70 nm on an ultra-thin slicer (Leica UC7, Leica, German). Sections were then double stained with 3% uranium acetate-lead citrate. Next, the morphology of the syncytiotrophoblast cells were observed under a transmission electron microscope (HT7700, HITACHI, Japan) and determined by ImageJ software.

### Quantitative real-time PCR (qRT-PCR)

For each sample, 50 mg of tissue were ground in a mortar and then mixed vigorously with 1 ml of Trizol (Thermo Fisher Scientific Co., Ltd.). Samples were then centrifuged at 12000 rpm for 5 min and the supernatant transferred into a new 1.5 ml microcentrifuge tube and mixed with 200 μl of chloroform. Samples were then centrifuged at 12,000 rpm for 15 min at 4 °C and the supernatant was transferred into a new 1.5 ml microcentrifuge. Samples were then mixed with the same volume of isopropanol and inverted several times to mix. Samples were then placed at − 20 °C for 30 min. Then, samples were centrifuged at 12000 rpm for 10 min at 4 °C; the supernatant was then discarded and 1 ml of 75% ethanol (anhydrous ethanol: dd H_2_O = 3:1) was added. The microcentrifuge tube was then oscillated to encourage precipitation and was then centrifuged at 8000 rpm at 4 °C. The supernatant was discarded and the pellet (total RNA) was dried at room temperature for 5–10 min. Then, the total RNA was resuspended in 30 µl of dd H_2_O water. The total RNA concentration and OD260/280 ratio were then determined with an ultra-micro spectrophotometer (Thermo Scientific, American).

We used a TB Green0R Premix Ex TaqTM (Tli RNaseH Plus) and PrimeScriptTM RT Reagent Kit (Perfect Real Time) that was purchased from TaKaRa Company (Japan). Using total RNA as a template, we prepared the reverse transcription reaction solution and qRT-PCR reaction solution, in accordance with the manufacturer’s instructions. Primer details are given in Table 2. qRT-PCR reaction system is given in Table 3. We used the levels of β-actin mRNA as a control; the relative expression levels of mRNA in placental tissues and cells were calculated by the 2^−∆∆CT^ method. The primers used in this experiment were synthesized by Fuzhou SunYa Biology Co., Ltd.

**Table 2:** Primer information in placental tissue experiment.

Objective gene	Primer sequence
β-actin	F ATCATGTTTGAGACCTTCAACA R CATCTCTTGCTCGAAGTCCA
DJ-1	F GTAGCCGTGATGTGGTCATTT R CTGTGCGCCCAGATTACCT
Nrf2	F AAGTGGAAGAGCTAGATAGTGCC R CCTGAGATGGTGACAAGGGTT
GPX4	F CAGGAGCCAGGFAGTAACGA R GCCCTTGGTGGATCTTCA

**Table 3:** qRT-PCR reaction system.

Reagent	Volume(μL)
SYBR Premix Ex Taq	30.0
PCR Forward Primer (10 μM)	1.2
PCR Reverse Primer (10 μM)	1.2
cDNA (< 100 ng)	6.0
ROX Reference Dye (50 ×)	1.2
dH_2_O	20.4
Total	60.0

### Western blotting

For western blotting, 50–100 mg of sample was mixed with 500 μl and 1000 μl of RIPA lysis buffer (Meilun Biotechnology Co., Ltd., Suzhou, China) and PMSF (1 mM) (Meilun Biotechnology Co., Ltd., Suzhou, China). Then, samples were centrifuged at 12000 rpm and 4 °C for 10 min. The protein concentration of each protein lysate (the supernatant) was then determined with a BCA Protein Assay Kit (Meilun Biotechnology Co., Ltd., Suzhou, China). Then, we took the same protein content from each sample (50–100 μg) and added the same volume of 5 × protein buffer (Meilun Biotechnology Co., Ltd., Suzhou, China). Then, protein samples were boiled for 10 min in boiling water.

SDS-PAGE (Beyotime Biotechnology Co., Ltd., Shanghai, China) gels were prepared in accordance with the manufacturer’s guidelines and samples loaded (V = 50 μg/C/$\frac{4}{5}$) and separated. Separated proteins were then transferred to PVDF membranes and cut the membranes into suitable size according to protein ladder and the molecular weight of target protein. Incubated with antibodies raised against β-actin (Immunoway, 1:1000, 4 °C), DJ-1 Polyclonal Antibody (Immunoway, 1:1000, 4 °C), Nrf2 Polyclonal Antibody (Immunoway, 1:1000, 4 °C), and GPX4 Polyclonal Antibody (Immunoway, 1:1000, 4 °C) in accordance with the manufacturer’s instructions. Incubated the membranes in the secondary antibody solution of HRP Goat Anti Mouse IgG (H+L) (Immunoway, 1:1000) at room temperature for 2 h. The optical densities of the positive binding bands were used meilunbio® FGSuper Sensitive ECL Luminescence Reagent (Dalian Melun Biotechnology Co., Ltd.) then quantified by Image Lab analysis software (National Institutes of Health).

### Immunohistochemistry

Placenta tissue was sectioned into 3 mm blocks and put the bloks into xylene I (Shanghai Lingfeng Chemical Reagent Co., LTD, 1330-20-7) for 15 min–xylene II for 15 min–xylene III for 15 min–absolute ethanol I (China National Pharmaceutical Group Chemical Reagent Co., LTD, 100092683) for 5 min–absolute ethanol II for 5 min–85% alcohol for 5 min–75% alcohol for 5 min–rinse in distilled water. The blocks are placed in a repair box filled with citric acid (PH6.0) antigen retrieval buffer (Servicebio, G1202) for antigen retrieval in a microwave oven, heated on medium power for 8 min until boiling, then turned off the microwave oven, kept warm for 8 min and then transferred to medium–low power for heating 7 min. During this process, excessive evaporation of buffer should be prevented and the sections should not be allowed to dry. To cool to room temperature before proceeding, the sections are placed in PBS (PH7.4) (Servicebio, G0002) and shaken on the decolorization shaker 3 times for 5 min each. The blocks are placed in 3% hydrogen peroxide (Pharmaceutical Group Chemical Reagent Co., LTD, 10011208) and incubated at room temperature in darkness for 25 min. The blocks are placed in PBS (PH7.4) and shaken on a decolorizing shaper 3 times for 5 min each. 3%BSA (Servicebio, G5001) was added to the circle to evenly cover the tissue, and the tissues are sealed for 30 min at room temperature. The sealing solution is gently removed, the DJ-1 Polyclonal Antibody (Immunoway) prepared with PBS (PH7.4) in a certain proportion is added to the blocks, and the blocks are placed flat in a wet box and incubated overnight at 4 °C. The blocks are placed in PBS (PH7.4) and washed by shaking on the decolorizing shaker 3 times for 5 min each. After the blocks are slightly shaken and dried, the tissues are covered with HRP Goat Anti Mouse IgG (H+L) (Immunoway) (HRP labeled) from the corresponding species of primary antibody and incubated at room temperature for 50 min. The blocks are placed in PBS (PH7.4) and shaken on the decoloring shaker 3 times for 5 min each. DAB (Servicebio, G1211) color developing solution newly prepared is added in the circle after the blocks are slightly dried. The positive is brownish yellow. Rinse the blocks with tap water to stop the reaction. The blocks are counterstained with hematoxylin stain solution (Servicebio, G1004) for about 3 min; washed with tap water; differentiated with hematoxylin differentiation solution (Servicebio, G1309) for several seconds; washed with tap water; treated with hematoxylin returning blue solution (Servicebio, G1340); washed with running water. Place the block in 75% alcohol for 5 min–85% alcohol for 5 min–absolute ethanol I 5 min–anhydrous ethanol II 5 min–n-butanol 5 min–xylene I 5 min, dehydrated and transparent, remove the blocks from xylene and let them dry slightly, then mount the blocks with neutral gum (Pharmaceutical Group Chemical Reagent Co., LTD, 10004160). Stained sections were analyzed using a positive white light photographic microscope and Image-Pro Plus 6.0 analysis software (Media Cybemetics, U.S.A).

### Cell treatment

The BeWo cells used in this study were purchased from Procell Life Science & Technology Co., Ltd. (Wuhan, China.) and cultured at 37 °C in 5% CO_2_. DJ-1+/+, DJ-1+/+ negative control, and DJ-1−/− and DJ-1−/− negative control viruses were purchased from Hanheng Biotechnology (Shanghai, China) Co., Ltd. The lentiviral vector siRNA sequence for DJ-1 interference was: 5-AGCTCCACTTTTTCTTAAGACTAGACTA-3. The lentiviral vector sequence for DJ-1 overexpression was as follows: 5-ATGGCTTCCAAAAGAGCTCTGGTCATCCTGGCT AAAGGAGCAGAGGAAATGGAGACGGTCATCCCTGTAGATGTCATGAGGCGAGCTGGGATTAAGGTCACCGTTGCAGGCCTGGCTGGAAAAGACCCAGTACAGTGTAGCCGTGATGTGGTCATTTGTCCTGATGCCAGCCTTGAAGATGCAAAAAAAGAGGGACCATATGATGTGGTGGTTCTACCAGGAGGTAATCTGGGCGCACAGAATTTATCTGAGTCTGCTGCTGTGAAGGAGATACTGAAGGAGCAGGAAAACCGGAAGGGCCTGATAGCCGCCATCTGTGCAGGTCCTACTGCTCTGTTGGCTCATGAAATAGGTTTTGGAAGTAAAGTTACAACACACCCTCTTGCTAAAGACAAAATGATGAATGGAGGTCATTACACCTACTCTGAGAATCGTGTGGAAAAAGACGGCCTGATTCTTACAAGCCGGGGGCCTGGGACCAGCTTCGAGTTTGCGCTTGCAATTGTTGAAGCCCTGAATGGCAAGGAGGTGGCGGCTCAAGTGAAGGCTCCACTTGTTCTTAAAGACTAG-3. The negative control Scramble lentivirus vector was an empty vector that was devoid of a functional sequence. The transfection of BeWo cells with lentiviral constructs was carried out according to the manufacturer’s instructions. According to the pre-set MOI value and virus titer, the dose of virus solution should be added as follows: virus volume (μl) = MOI × number of cells/virus titer (TU/ml) × 1000. The cells were cultured in six-hole plate (1.2 × 10^6^/cell) in the half complete volume culture medium with the same volume of virus solution for 4 h. Replenish the remaining volume of culture medium. After the virus was treated with the cells for 24 h, the fresh and complete culture medium was changed and the culture continued for 48 h. Transfection efficiency was evaluated by fluorescence inversion microscopy (Nikon ECLIPSE Ts2R-FL).

Complete medium was prepared by mixing 85% Ham's F-12K basic medium (Procell Life Science & Technology Co., Ltd.) with 13% fetal bovine serum standard (NEWZERUM) and 2% penicillin/streptomycin solution (Meilun Biotechnology Co., Ltd.). Ferroptosis inducer RSL3 stock solution was prepared by dissolving 5 mg of RSL3 powder (APExBio) in 11.3410 ml of DMSO (Suzhou Meilun Biotechnology Co., Ltd.); this prepared 1 mM of stock solution, which was then stored at -20 °C. Next, we prepared a working solution for the ferroptosis inducer RSL3 by diluting the RSL3 stock solution to concentrations of 0, 2, 4, 6, 8, 10 μM with complete medium. The working solution for the ferroptosis inhibitor Fer-1 was prepared by diluting Fer-1 stock solution (APExBio) to a concentration of 2.5 μM with complete medium. Puromycin solution (10 mg/ml) (Dalian Meilun Biotechnology Co., Ltd.) was diluted to a concentration of 0.5 μg/ml with complete medium.

### Determination of LDH concentration

The LDH activity test kit used in this experiment was purchased from Beijing Solebao Biotechnology Co., Ltd. Diluted the 100 μL standard solution to 1, 0.5, 0.25, 0.125, 0 μmol/mL and made the standard curve with 2, 1, 0.5, 0.25, 0.125, 0 μmol/mol. LDH concentrations were then determined in accordance with Table 4. The absorbance value of 450 nm was measured in microwell plate spectrophotometer (BioTek Epoch). The standard curve is made according to the measured value and concentration of the standard tube, y (μmol/mL) is the standard concentration and x is the absorbance. LDH (U/10^4^cell) = 0.133y.

**Table 4:** LDH reaction system.

Reagent name (μL)	Measuring tube	Control tube	Standard tube
Sample	10	10	–
Standard solution	–	–	10
Reagent1	50	50	50
Reagent2	10	–	–
ddH_2_O	–	10	10
Mixing well and bathing at 37 °C for 15 min			
Reagent3	50	50	50
Mixing well and bathing at 37 °C for 15 min			
Reagent4	150	150	150

### Quantitative Real-time PCR (qRT-PCR)

The specific operation was the same as 2.1.1. Primer details are given in Table 5.

**Table 5:** Primer information in cell experiment.

Objective gene	Primer sequence
β-actin	F ATCATGTTTGAGACCTTCAACA R CATCTCTTGCTCGAAGTCCA
DJ-1	F TGATGCCAGCCTTGAAGATG R TCACAGCAGCAGACTCAGATA
Nrf2	F AAGTGGAAGAGCTAGATAGTGCC R CCTGAGATGGTGACAAGGGTT
GPX4	F CAGGAGCCAGGFAGTAACGA R GCCCTTGGTGGATCTTCA

### Statistical analysis

Data were statistically analyzed by the SPSS version 19.0 software package. The measurement data were expressed as mean ± standard deviation (SD). Differences among groups were compared by single factor analysis of variance (ANOVA), the type of post hoc test used LSD and comparison between two groups was conducted by the independent sample t-test. α was 0.05 and P < 0.05 was statistically significant.

### Ethical statement

The Ethics Committee of Fujian Maternity and Child Health Hospital approved all protocols (Reference: 2021KLRD631).

## Results

### Baseline characteristics of the women included in this analysis

There was no significant difference in maternal age (P = 0.252), gestational (G)(P = 0.073), parity (P)(P = 0.281) and diastolic pressure (DP)(P = 0.248) when compared between groups. The gestational age in the PE group was significantly lower than in the control group (P < 0.001). The prenatal BMI in the PE group was significantly higher than in the control group (P = 0.016). Systolic pressure (SP) in the PE group was significantly higher than in the control group (P = 0.025). (Table 6).

**Table 6:** Baseline characteristics of the women included in this analysis.

	Control group (n = 30)	PE group (n = 30)
Maternal age	31.17 ± 3.37	32.73 ± 4.49
Gestational age	38.50 ± 1.01	35.07 ± 3.08^a,^
G	2.57 ± 1.33	2.57 ± 2.10
P	0.77 ± 0.57	0.53 ± 0.57
Prenatal BMI(kg/m^2^)	26.90 ± 2.84	29.09 ± 4.66^a^
SP(mmHg)	116.13 ± 8.14	155.03 ± 13.49^a^
DP(mmHg)	75.77 ± 7.72	99.63 ± 10.31

^a^Compared with the control group P < 0.05. Comparison between two groups was conducted by the independent sample t-test.

### Ferroptosis markers in preeclamptic placentae

#### The concentration of MDA in placental tissue

**Figure 1:**
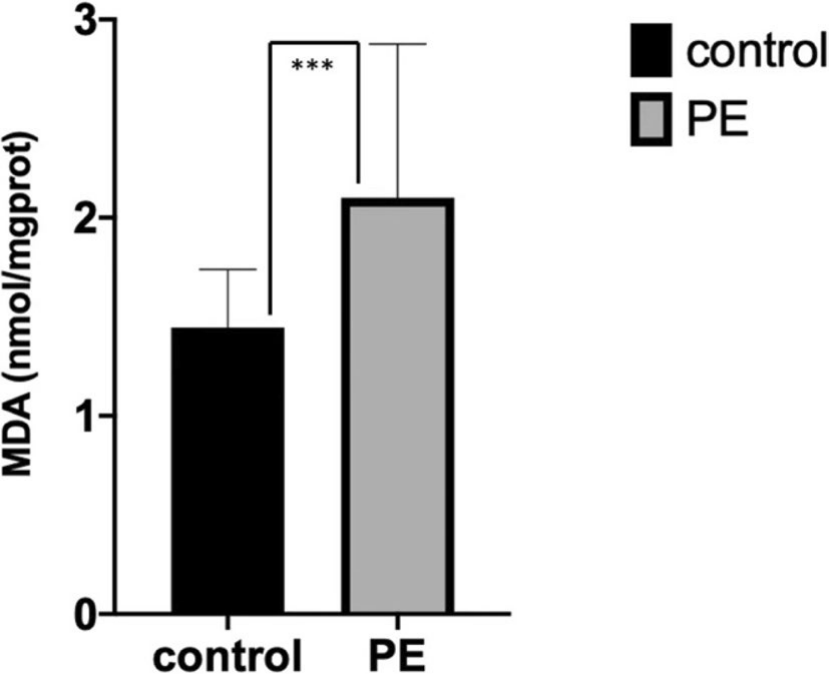
Concentration of MDA in placental tissue from normal and preeclamptic pregnancies. Concentration of MDA was significantly increased in PE group (P < 0.001). Comparison between two groups was conducted by the independent sample t-test.

The concentration of MDA in the PE group was significantly higher than that in the control group [control vs. PE = (1.44 ± 0.29) (nmol/mgprot) vs. (2.10 ± 0.78) (nmol/mgprot), P < 0.001] (Fig. 1).

#### Morphology of placental trophoblasts

**Figure 2:**
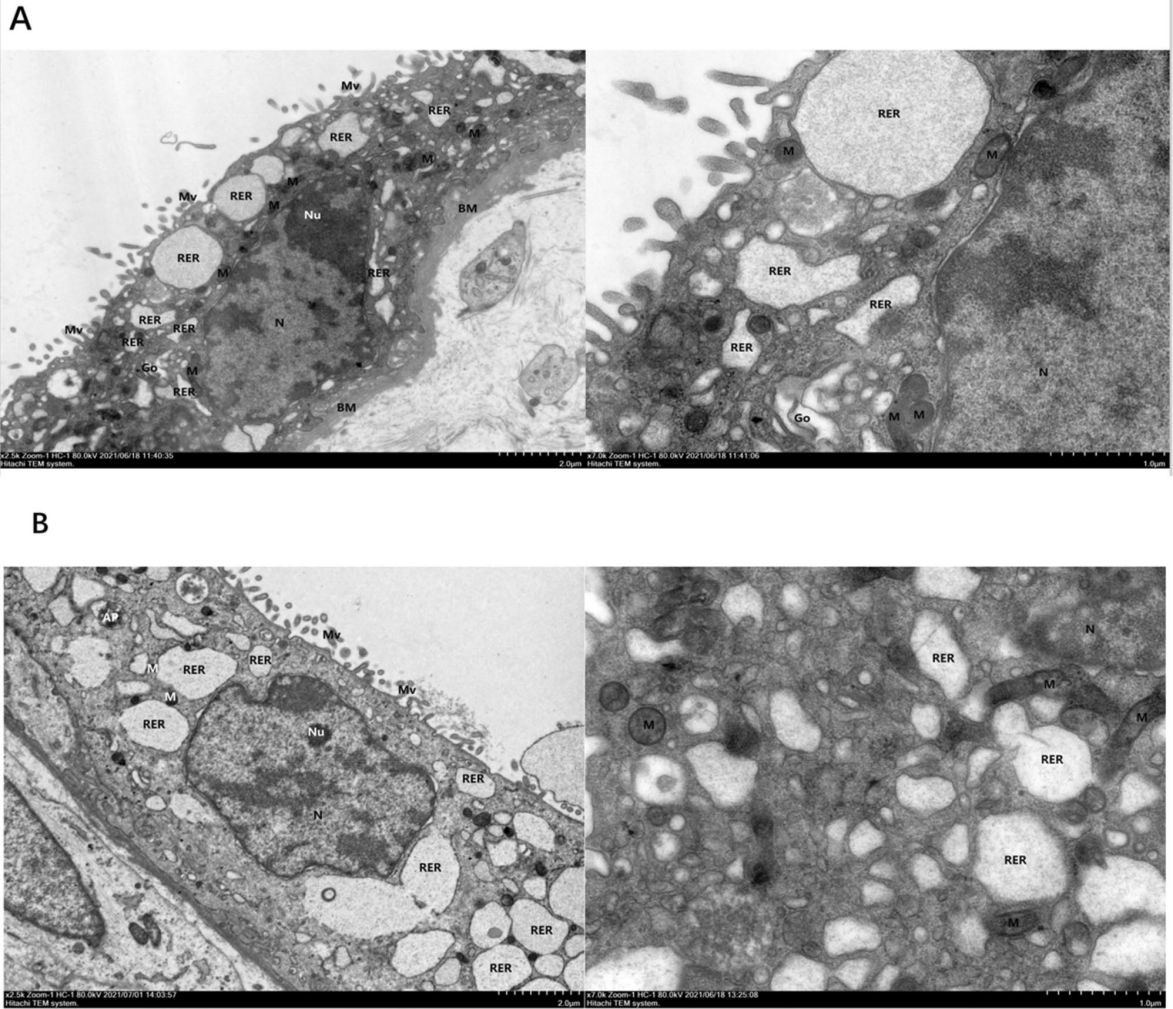
Morphology of placental trophoblasts from normal and preeclamptic pregnancies. (**A**) Control group, (**B**) PE group. The phenomenon of ferroptosis in group (**B**) was more serious than that in group (**A**).

Figure 2A shows that in the control group, the syncytiotrophoblast cells in placental tissue showed slight evidence of pyknosis, the basement membrane (BM) was continuous and intact, thickness was uniform, the tight junctions between cells were reduced, and the local intercellular space was slightly widened. Cell membranes were intact with abundant microvilli (Mv) on their surface. Cells also exhibited a high electron density, abundant organelles, partial swelling, irregular shaped nuclei (N), local depression, a complete nuclear membrane, local widening of the perinuclear space, a large amount of heterochromatin, and nucleolar (Nu) edge shift. There was a high number of mitochondria (M) with an acceptable and intact structure, and a parallel crest. The rough endoplasmic reticulum (RER) was obviously dilated and degranulated; there was evidence of more floc accumulation and endoplasmic reticulum retention. The Golgi apparatus (Go) was slightly hypertrophic and the capsule was dilated; there was no typical autophagy structure in the visual field (magnification × 2500).

As can be seen from Fig. 2B, the syncytiotrophoblast cells in placental tissue from the PE group showed obvious pyknosis. The tight junctions between cells were reduced, and the local intercellular space had widened slightly. The cell membranes were intact with abundant microvilli (Mv) on their surface. Intracellular electron density was high and there were many swollen organelles. Nuclei (N) showed irregular pyknosis; the nuclear membrane was intact, the perinuclear space was normal, and there was a greater amount of heterochromatin than the control group. There was an abundance of mitochondria (M); these were mostly swollen. The mitochondrial matrix was lighter in the membrane, and the crest had reduced and disappeared. The rough endoplasmic reticulum (RER) was obviously dilated, degranulated, and vacuolated. The membrane of the RER was damaged and disintegrated in severe cases; there was no typical autophagy evident in the visual field (magnification × 2500).

Images of syncytiotrophoblast cells in the two groups showed varying degrees of injury. The cells in the control group showed mild pyknosis, slight edema, a high intracellular electron density, abundant organelles, and partial swelling. Cells in the PE group showed relatively severe ferroptosis injuries, obvious pyknosis and cell shrinkage, high intracellular electron density, abundant organelles, and obvious swelling.

### Expression levels of *DJ-1* and *Nrf2/GPX4* signal pathway in placental tissue

#### Expression levels of *DJ-1* in placental tissue

**Figure 3:**
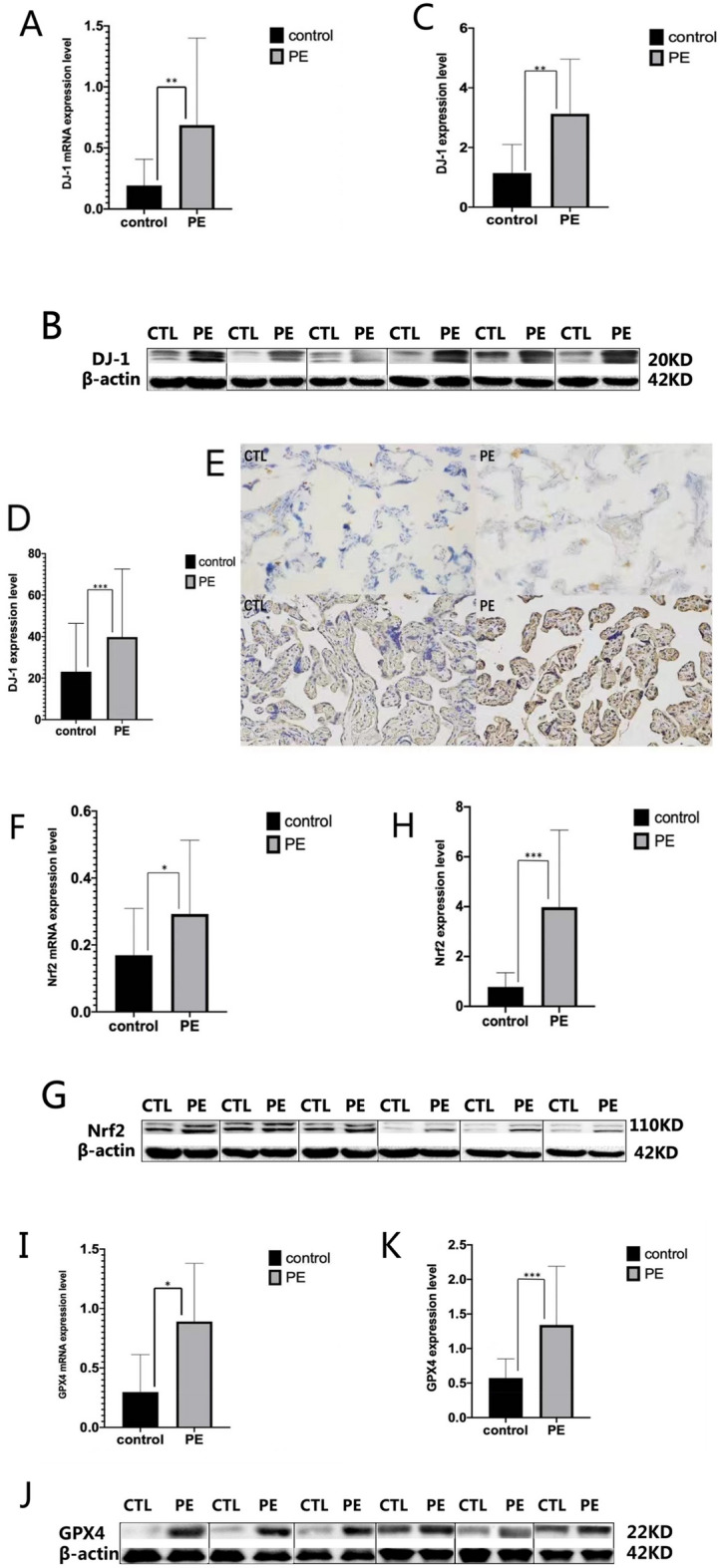
*DJ-1* and *Nrf2/GPX4* signal pathway expression levels in placental tissue from normal and preeclamptic pregnancies. (**A**,**F**,**I**) In reverse transcriptase-polymerase chain reaction (RT-PCR), expression level of DJ-1, Nrf2 and GPX4 mRNA was significantly increased in PE group (P_1_ = 0.005, P_2_ = 0.022, P_3_ = 0.029). (**B**,**C**,**G**,**H**,**J**,**K**) In Western Blot, expression level of DJ-1, Nrf2 and GPX4 protein was significantly increased in PE group (P_1_ = 0.005, P_2_ < 0.001, P_3_ < 0.001). (**D**,**E**) In immunohistochemistry, expression level of DJ-1 protein was significantly increased in PE group (p < 0.001). Comparison between two groups was conducted by the independent sample t-test.

The expression levels of *DJ-1* mRNA in the PE group were significantly higher than those in the control group [control vs. PE = (0.19 ± 0.21) vs. (0.69 ± 0.71), P = 0.005] (Fig. 3A). The expression levels of *DJ-1* protein in the PE group were significantly higher than those in the control group [control vs. PE = (1.14 ± 0.96) vs. (3.13 ± 1.83), P = 0.005] (Fig. 3B,C).

The proportion (%) of *DJ-1* positive cells in the PE group was significantly higher than that in the control group [control vs. PE = (23.11 ± 23.28) vs. (39.80 ± 32.81), P < 0.001] (magnification × 200) (Fig. 3D,E).

#### Expression levels of *Nrf2* in placental tissue

The expression levels of *Nrf2* mRNA in the PE group were significantly higher than those of the control group[control vs. PE = (0.17 ± 0.14) vs. (0.29 ± 0.22), P = 0.022] (Fig. 3F). The expression levels of *Nrf2* protein in the PE group were significantly higher than the control group[control vs. PE = (0.78 ± 0.57) vs. (3.98 ± 3.09), P < 0.001] (Fig. 3G,H).

#### Expression levels of *GPX4* in placental tissue

The expression levels of *GPX4* mRNA in the PE group were significantly higher than those in the control group[control vs. PE = (0.30 ± 0.32) vs. (0.89 ± 0.49), P = 0.029] (Fig. 3I). The expression levels of *GPX4* protein in the PE group were significantly higher than those in the control group[control vs. PE = (0.57 ± 0.28) vs. (1.34 ± 0.85), P < 0.001]. (Fig. 3J,K).

### Correlations of DJ-1 protein expression levels with the Nrf2/GPX4 signaling pathway protein expression levels and MDA concentrations in placental tissue

**Figure 4:**
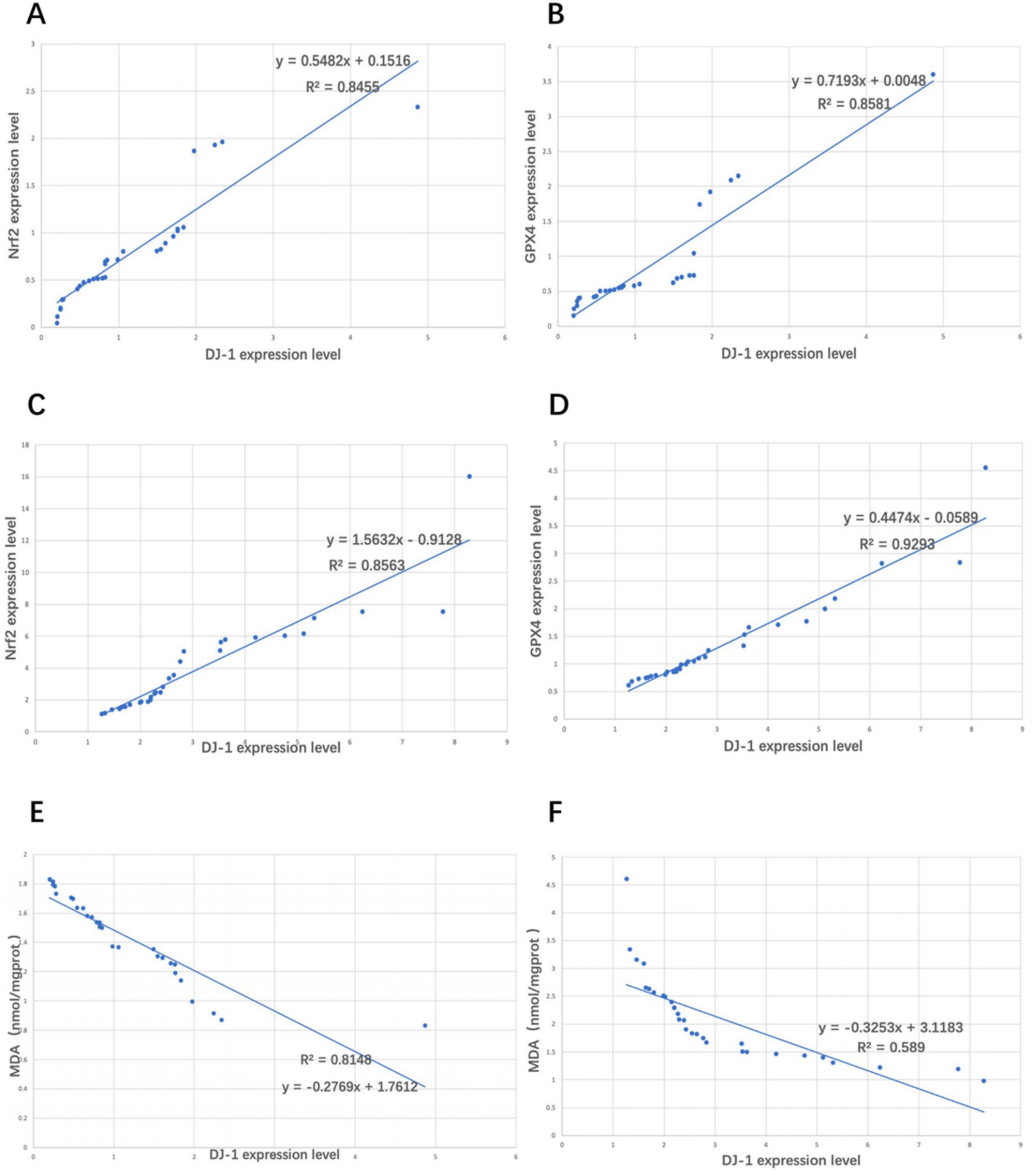
Correlations of DJ-1 protein expression levels with the Nrf2/GPX4 signaling pathway protein expression levels and MDA concentrations in each group. (**A**,**B**,**E**) The expression level of DJ-1 protein in the control group was positively correlated with the expression levels of the Nrf2/GPX4 signaling pathway protein, and negatively correlated with the concentrations of MDA (P < 0.001). (**C**,**D**,**F**) The expression level of DJ-1 protein in the PE group was positively correlated with the expression levels of the Nrf2/GPX4 signaling pathway protein, and negatively correlated with the concentrations of MDA (P < 0.001).

The expression levels of DJ-1 protein in the control group (Fig. 4A,B) and the PE group (Fig. 4C,D) were positively correlated with the expression levels of the Nrf2/GPX4 signaling pathway protein (P < 0.001), and negatively correlated with the concentrations of MDA (P < 0.001) (Fig. 4E,F) (Table7).

**Table 7:** Correlations of DJ-1 protein expression levels with the Nrf2/GPX4 signaling pathway protein expression levels and MDA concentrations in each group (r value).

Group	MDA	Nrf2	GPX4
Control	− 0.90 ^a^	0.92 ^a^	0.93 ^a^
PE	− 0.77 ^a^	0.93 ^a^	0.96 ^a^

^a^Compared with the DJ-1 group P < 0.001.

### Effects of the ferroptosis inducer RSL3 and the ferroptosis inhibitor Fer-1 on the rate of LDH release from BeWo cells

#### Effects of different concentrations of the ferroptosis inducer RSL3 on the rate of LDH release from BeWo cells

**Figure 5:**
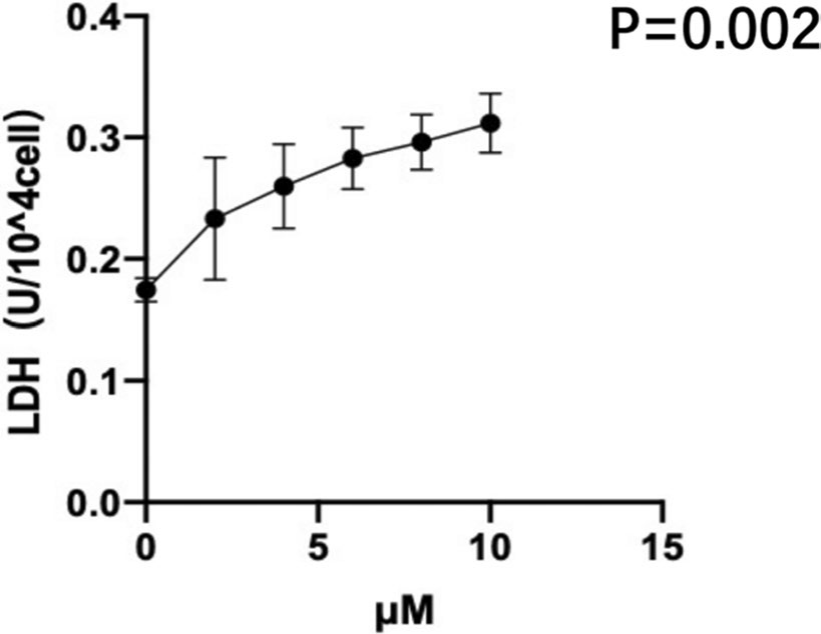
Effects of different concentrations of RSL3 on the rate of LDH release from BeWo cells (biological replicates number: 1). With the gradual increase of the ferroptosis inducer RSL3 concentration, the mortality rate of BeWo cells increased significantly. Differences among groups were compared by single factor analysis of variance (ANOVA).

After 24 h of cell culture, increasing concentrations of the ferroptosis inducer RSL3 (0, 2, 4, 6, 8, 10 μM) led to an increase in the concentrations of LDH in the supernatant of BeWo cells (biological replicates number: 1); this also led to an increase in the mortality rate of BeWo cells (P = 0.002) (Table 8 and Fig. 5).

**Table 8:** Effects of different concentrations of RSL3 on the rate of LDH release from BeWo cells (${{\overline{\text{X}} + S}}$).

RSL3(μM)	0	2	4	6	8	10
LDH(U/10^4^cell)	0.17 ± 0.01	0.23 ± 0.05^a^	0.26 ± 0.03^a^	0.28 ± 0.03^a^	0.30 ± 0.02^a^	0.31 ± 0.02^a^

^a^Compared with 0 μM group P < 0.05.

#### Effects of the ferroptosis inducer RSL3 (10 μM) and the ferroptosis inhibitor Fer-1 (2.5 μM) on the rate of LDH release from BeWo cells

**Figure 6:**
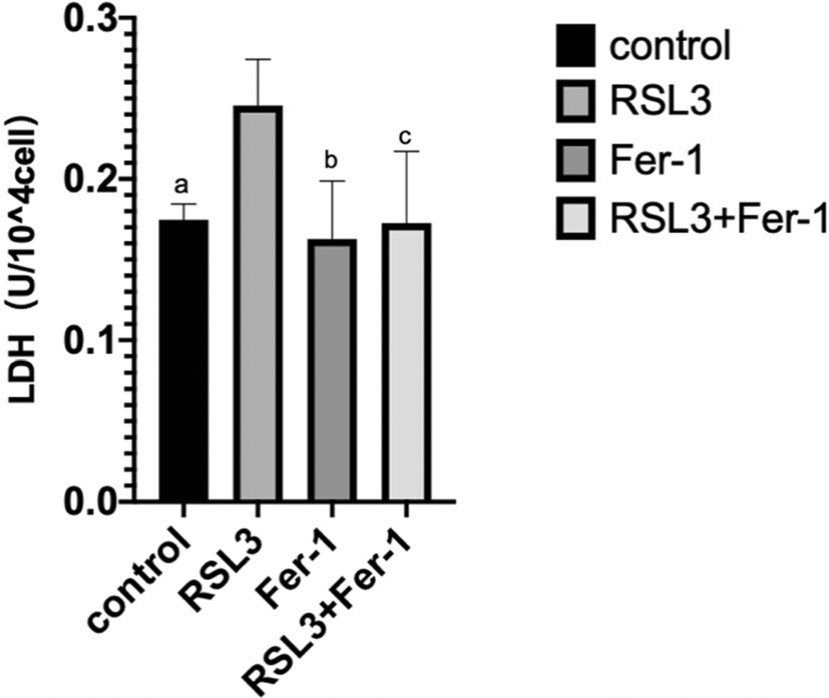
Effects of RSL3 and Fer-1 on the rate of LDH release from BeWo cells (biological replicates number: 1). (**a**–**c)** Compared to the RSL3 group, the mortality rate of BeWo cells in the control group, the Fer-1 group and the RSL3 + Fer-1 group decreased significantly(P_a_ = 0.018, P_b_ = 0.005, P_c_ = 0.011). Differences among groups were compared by single factor analysis of variance (ANOVA) the type of post hoc test used LSD.

After 24 h of cell culture, the concentration of LDH in the supernatant of BeWo cells (biological replicates number: 1) in the control, Fer-1 and RSL3 + Fer-1 group was significantly lower than that in the RSL3 group (Fig. 6) [RSL3 vs. control = (0.25 ± 0.03) vs. (0.17 ± 0.01), P = 0.018] [RSL3 vs. Fer-1 = (0.25 ± 0.03) vs. (0.16 ± 0.04), P = 0.005] [RSL3 vs. RSL3 + Fer-1 = (0.25 ± 0.03) vs. (0.17 ± 0.04), P = 0.011]; in other words, the mortality rate of BeWo cells decreased significantly. There was no significant change in the concentration of LDH in the supernatant of BeWo cells among the control group, the RSL3 + Fer-1 group and the Fer-1 group [control vs. RSL3 + Fer-1 = (0.17 ± 0.01) vs. (0.17 ± 0.04), P = 0.942] [control vs. Fer-1 = (0.17 ± 0.01) vs. (0.16 ± 0.04), P = 0.651] [Fer-1 vs. RSL3 + Fer-1 = (0.16 ± 0.04) vs. (0.17 ± 0.04), P = 0.710]. In other words, there was no significant change in the mortality rate in BeWo cells. Combined with the above results of RSL3 concentration gradient experiment, it is suggested that BeWo cells are indeed sensitive to ferroptosis. It is speculated that trophoblast ferroptosis plays an important role in the pathogenesis of PE.

### Transfection efficiency of DJ-1 lentivirus in BeWo cells

**Figure 7:**
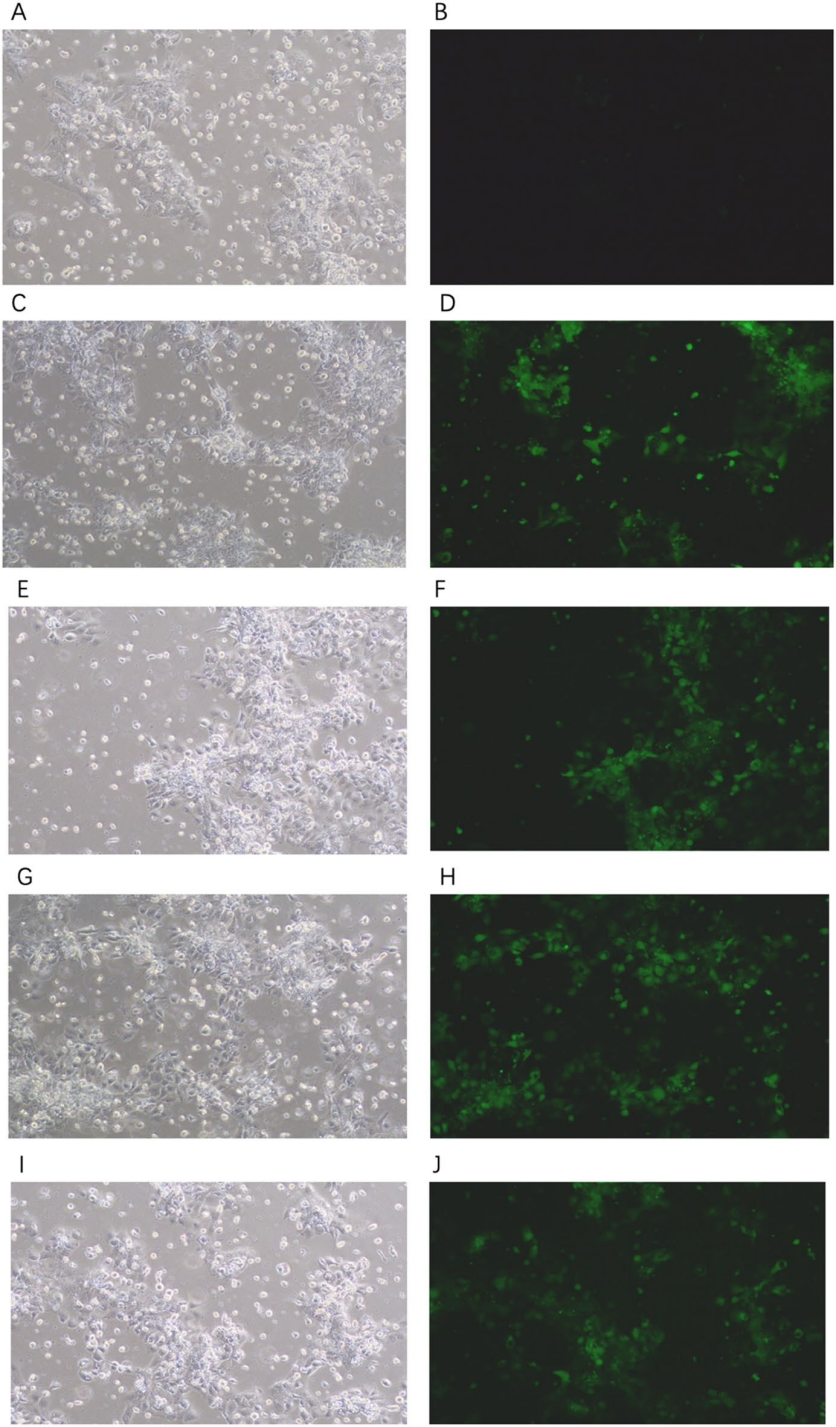
Different groups transfection efficiency. (**A**,**B**) The blank group; (**C**,**D**) the *DJ-1* overexpression group (expressed in *DJ-1*+*/*+); (**E**,**F**) the *DJ-1*+*/*+ negative control group; (**G**,**H**) the *DJ-1* inhibited expression group (expressed in *DJ-1*−/−); (**I**,**J**) the *DJ-1*−/− negative control group. (**A**,**C**,**E**,**G**,**I**) the bright-field images; (**B**,**D**,**F**,**H**,**J**) the dark-field images (biological replicates number: 2).

Figure 7 show transfection efficiency data. In the blank group, when compared to the bright-field images, the fluorescent cell ratio of the dark-field images was almost 0 (Fig. 7A,B). Compared with the bright-field images, the fluorescent cell ratio of the dark-field images in the *DJ-1* overexpression group (expressed in *DJ-1*+*/*+) (Fig. 7C,D), the DJ-1+/+ negative control group (Fig. 7E,F), the *DJ-1* inhibited expression group (expressed in *DJ-1*−/−) (Fig. 7G,H), and the *DJ-1*−/− negative control group (Fig. 7I,J), ranged from 60 to 70% (magnification × 200) (biological replicates number: 2).

### Expression levels of *DJ-1* and *Nrf2/GPX4* signal pathway mRNA in transfected BeWo cells

#### Expression levels of *DJ-1* mRNA in transfected BeWo cells

**Figure 8:**
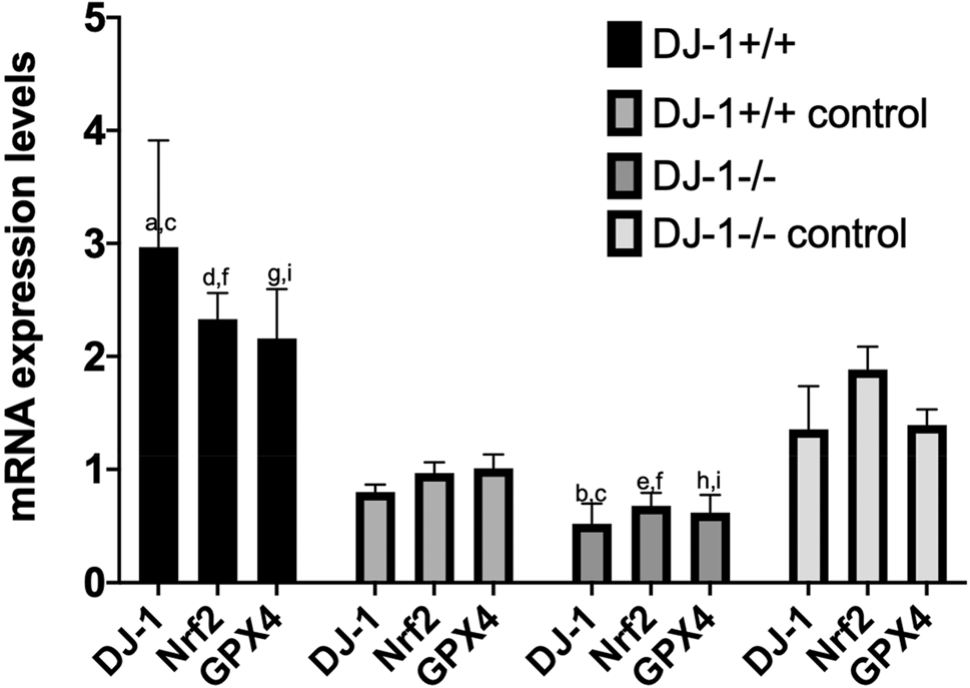
*DJ-1* and *Nrf2/GPX4* signal pathway mRNA expression levels in transfected BeWo cells. (**a**) Compared to the *DJ-1*+*/*+ negative control group, the expression level of *DJ-1* mRNA in BeWo cells from the *DJ-1*+*/*+ group was significantly higher (P < 0.001). (**b**) Compared to the *DJ-1*−/− negative control group, the expression level of *DJ-1* mRNA in BeWo cells from the *DJ-1*−/− group was significantly lower (P = 0.042). (**c**) Compared to the *DJ-1*−/− group, the expression level of *DJ-1* mRNA in BeWo cells from the *DJ-1*+*/*+ group was significantly higher (P < 0.001). (**d**) Compared to the *DJ-1*+*/*+ negative control group, the expression level of *Nrf2* mRNA in BeWo cells from the *DJ-1*+*/*+ group was significantly higher (P < 0.001). (**e**) Compared to the *DJ-1*−/− negative control group, the expression level of *Nrf2* mRNA in BeWo cells from the *DJ-1*−/− group was significantly lower (P < 0.001). (**f**) Compared to the *DJ-1*−/− group, the expression level of *Nrf2* mRNA in BeWo cells from the *DJ-1*+*/*+ group was significantly higher (P < 0.001). (**g**) Compared to the *DJ-1*+*/*+ negative control group, the expression level of *GPX4* mRNA in BeWo cells from the *DJ-1*+*/*+ group was significantly higher (P < 0.001). (**h**) Compared to the *DJ-1*−/− negative control group, the expression level of *GPX4* mRNA in BeWo cells from the *DJ-1*−/− group was significantly lower (P = 0.005). (**i**) Compared to the *DJ-1*−/− group, the expression level of *GPX4* mRNA in BeWo cells from the *DJ-1*+*/*+ group was significantly higher (P < 0.001). Differences among groups were compared by single factor analysis of variance (ANOVA) the type of post hoc test used LSD. (Biological replicates number: 2).

Figure 8 shows that the expression levels of *DJ-1* mRNA in BeWo cells from the *DJ-1*+*/*+ group were significantly higher than those in the *DJ-1*+*/*+ negative control group [*DJ-1*+*/*+ vs. *DJ-1*+*/*+ negative control = (2.97 ± 0.95) vs. (0.80 ± 0.07), P < 0.001]. The expression levels of *DJ-1* mRNA in BeWo cells from the *DJ-1*−/− group were significantly lower than those in the *DJ-1*−/− negative control group [*DJ-1*−/− vs. *DJ-1*−/− negative control = (0.52 ± 0.18) vs. (1.35 ± 0.38), P = 0.042]. The expression levels of *DJ-1* mRNA in BeWo cells from the *DJ-1*+*/*+ group were significantly higher than those in the *DJ-1*−/− group[*DJ-1*+*/*+ vs. *DJ-1*−/− = (2.97 ± 0.95) vs. (0.52 ± 0.18), P < 0.001]. These results showed that the transfection effect of *DJ-1* lentivirus in BeWo cells was effective (biological replicates number: 2).

#### Expression levels of Nrf2 mRNA in transfected BeWo cells

The expression levels of *Nrf2* mRNA in BeWo cells from the *DJ-1*+*/*+ group were significantly higher than those in the *DJ-1*+*/*+ negative control group [*DJ-1*+*/*+ vs. *DJ-1*+*/*+ negative control = (2.33 ± 0.23) vs. (0.97 ± 0.10), P < 0.001] (Fig. 8). The expression levels of *Nrf2* mRNA in BeWo cells from the *DJ-1*−/− group were significantly lower than those in the *DJ-1*−/− negative control group[*DJ-1*−/− vs. *DJ-1*−/− negative control = (0.68 ± 0.12) vs. (1.88 ± 0.20), P < 0.001]. The expression levels of *Nrf2* mRNA in BeWo cells from the *DJ-1*+*/*+ group were significantly higher than those in the *DJ-1*−/− group [*DJ-1*+*/*+ vs. *DJ-1*−/− = (2.33 ± 0.23) vs. (0.68 ± 0.12), P < 0.001] (biological replicates number: 2).

#### Expression levels of GPX4 mRNA in transfected BeWo cells

The expression levels of *GPX4* mRNA in BeWo cells from the *DJ-1*+*/*+ group were significantly higher than those in the *DJ-1*+*/*+ negative control group [*DJ-1*+*/*+ vs. *DJ-1*+*/*+ negative control = (2.16 ± 0.44) vs. (1.01 ± 0.12), P < 0.001] (Fig. 8). The expression levels of *GPX4* mRNA in BeWo cells from the *DJ-1*−/− group were significantly lower than those in the *DJ-1*−/− negative control group[*DJ-1*−/− vs. *DJ-1*−/− negative control = (0.62 ± 0.16) vs. (1.39 ± 0.14), P = 0.005]. The expression levels of *GPX4* mRNA in BeWo cells from the *DJ-1*+*/*+ group were significantly higher than those in the *DJ-1*−/− group[*DJ-1*+*/*+ vs. *DJ-1*−/− = (2.16 ± 0.44) vs. (0.62 ± 0.16), P < 0.001] (biological replicates number: 2).

### Effect of the ferroptosis inducer RSL3 on the release rate of LDH in transfected BeWo cells

**Figure 9:**
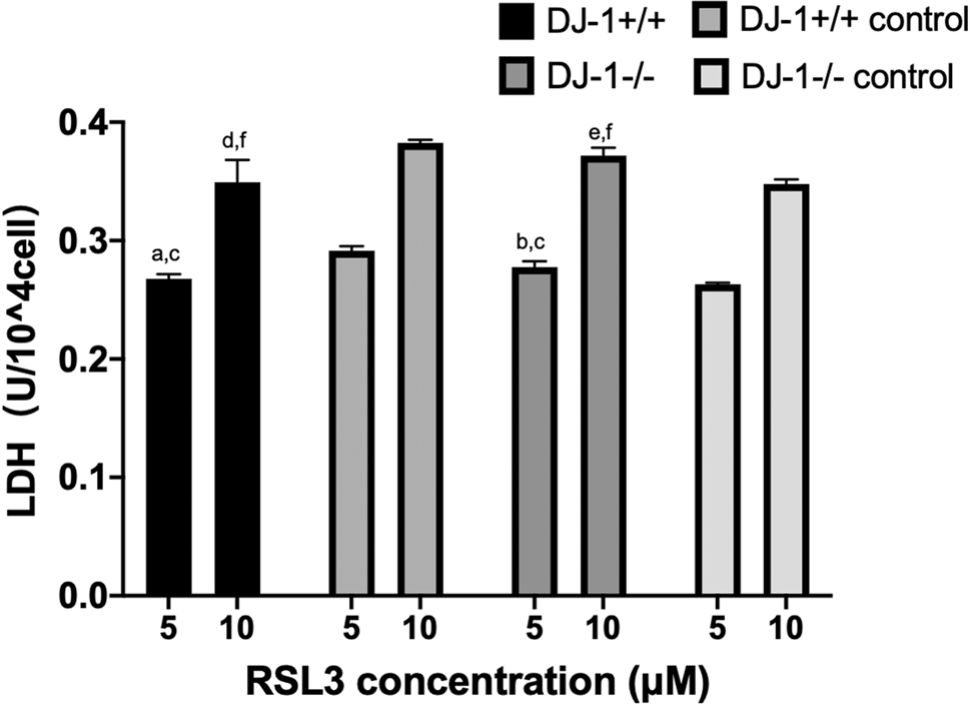
Effect of RSL3 on the release rate of LDH in transfected BeWo cells. (**a**–**c**) Effect of RSL3 (5 μM) on the release rate of LDH in transfected BeWo cells. (**a**) Compared to the *DJ-1*+*/*+ negative control group, the mortality rate of BeWo cells in the *DJ-1*+*/*+ group decreased significantly (P < 0.001). (**b**) Compared to the *DJ-1*−/− negative control group, the mortality rate of BeWo cells in the *DJ-1*−/− group increased significantly (P = 0.001). (**c**) Compared to the *DJ-1*−/− group, the mortality rate of BeWo cells in the *DJ-1*+*/*+ group decreased significantly (P = 0.012) (biological replicates number: 2). (**d**–**f**) Effect of RSL3 (10 μM) on the release rate of LDH in transfected BeWo cells. (**d**) Compared to the *DJ-1*+*/*+ negative control group, the mortality rate of BeWo cells in the *DJ-1*+*/*+ group decreased significantly (P = 0.004). (**e**) Compared to the *DJ-1*−/− negative control group, the mortality rate of BeWo cells in the *DJ-1*−/− group increased significantly (P = 0.021). (**f**) Compared to the *DJ-1*−/− group, the mortality rate of BeWo cells in the *DJ-1*+*/*+ group decreased significantly (P = 0.028) (biological replicates number: 2). Differences among groups were compared by single factor analysis of variance (ANOVA) the type of post hoc test used LSD.

When 5 μM of the ferroptosis inducer RSL3 was added for 24 h, we found that the LDH concentrations in BeWo cells from the *DJ-1*+*/*+ group were significantly lower than those in the *DJ-1*+*/*+ negative control group [*DJ-1*+*/*+ vs. *DJ-1*+*/*+ negative control = (0.27 ± 0.004) vs. (0.29 ± 0.004), P < 0.001] (Fig. 9). The concentrations of LDH in BeWo cells from the *DJ-1*−/− group were significantly higher than those in the *DJ-1*−/− negative control group[*DJ-1*−/− vs. *DJ-1*−/− negative control = (0.28 ± 0.005) vs. (0.26 ± 0.001), P = 0.001]. The LDH concentrations in BeWo cells from the *DJ-1*+*/*+ group were significantly lower than those in the *DJ-1*−/− group[*DJ-1*+*/*+ vs. *DJ-1*−/− = (0.27 ± 0.004) vs. (0.28 ± 0.005), P = 0.012] (biological replicates number: 2).

When 10 μM of the ferroptosis inducer RSL3 was added for 24 h, the LDH concentrations of BeWo cells in the *DJ-1*+*/*+ group were significantly lower than those in the *DJ-1*+*/*+ negative control group [*DJ-1*+*/*+ vs. *DJ-1*+*/*+ negative control = (0.35 ± 0.02) vs. (0.38 ± 0.003), P = 0.004] (Fig. 9). The LDH concentration of BeWo cells in the *DJ-1*−/− group were significantly higher than those in the *DJ-1*−/− negative control group[*DJ-1*−/− vs. *DJ-1*−/− negative control = (0.37 ± 0.01) vs. (0.35 ± 0.004), P = 0.021]. Finally, the LDH concentrations in BeWo cells from the *DJ-1*+*/*+ group were significantly lower than those in the *DJ-1*−/− group[*DJ-1*+*/*+ vs. *DJ-1*−/− = (0.35 ± 0.02) vs. (0.37 ± 0.01), P = 0.028] (biological replicates number: 2).

## Discussion

In this study, we found that ferroptosis is involved in the pathogenesis of PE and that DJ-1 plays a protective role in the pathogenesis of PE by regulating the Nrf2/GPX4 signaling pathway to mediate ferroptosis in trophoblast cells.

According to the "six-stage model" theory for PE^15^, a series of PE clinical symptoms (such as hypertension and albuminuria) begin to appear after four stages: poor maternal immune tolerance during embryo implantation; abnormal trophoblast invasion in the critical period of placental formation; maternal–fetal interface stress response, and excessive concentrations of placental-derived damage factors that invade the maternal blood circulation. Ferroptosis is a new form of regulatory cell death that was first reported by Dixon in 2012^5^. The abnormal of iron mediates the excessive accumulation of lipid peroxidation, which involves a variety of physiological and pathological processes, including cancer cell death, neurotoxicity, neurodegenerative diseases, acute renal failure, drug-induced hepatotoxicity, liver and heart ischemia/reperfusion injury and T cell immunity^4^.

Placental oxidative stress (OS) and lipotoxicity have been confirmed to be characteristic manifestations of placental dysfunction; there is also evidence to suggest that trophoblast cell lines are sensitive to ferroptosis^16^. Barneo-Caragol et al.^17^ reported that the ratio of lipid peroxidation to total antioxidant activity (TAA) in the placentas of pregnant women with early-onset PE was significantly higher than that in women with late-onset PE and healthy pregnant women. In addition, metabolic analyses of placental mitochondria showed that the levels of polyunsaturated fatty acid (PUFAs), and the degree of mitochondrial shrinkage, in pregnant women with severe PE were significantly higher than those in a control group with normal blood pressure^18^, thus confirming that ferroptosis plays a key role in the pathogenesis of PE. Zhang et al.^19^ demonstrated that the concentration of MDA, and the level of total Fe^2+^, in the placenta of PE rats were also significantly increased coincident with a significant reduction in the levels of GSH and the activity of GPX. Furthermore, supplementation with a ferroptosis inhibitor was shown to alleviate the symptoms of PE in rats. There was also a significant increase in the concentration of MDA in the mitochondrial membrane, the extent of mitochondrial permeability transition pore (MPTP) opening, and the expression levels of mtDNA in the placentas of pregnant women with PE, thus confirmed the existence of ferroptosis;^20^ these findings were consistent with our present experimental results.

Mitochondria are representative regulatory cell death (RCD) executive organelles, which function by releasing or recruiting specific cell death-promoting factors, including ferroptosis^21^. Different from the mitochondrial swelling of traditional apoptosis, necrosis and autophagy, electron microscopy showed that the mitochondrial membrane of ferroptosis cells was dense, the cristae decreased or even disappeared, but most of the nuclei were normal^22^. In this study, it was found that the mitochondria of PE trophoblast showed not only the characteristics of ferroptosis, including the weakening of intramembrane matrix, the decrease and disappearance of crest, but also the morphological manifestation opposite to that of typical ferroptosis, that is, volume swelling. Because PE is a complex pregnancy-related disease caused by many factors, many studies have shown that apoptosis and necrosis can also mediate the pathogenesis of PE^23^, so we reasonably speculate that the complex morphological changes of mitochondria are the result of different ways of cell death. This study also found that there were some depressions and heterochromatin damage in trophoblast nuclei in both PE group and control group. This seems to be inconsistent with the previous literature that the nuclear membrane of ferroptosis cells is normal^24^. However, it should be noted that even in normal pregnancy, placental tissue will experience varying degrees of OS damage caused by hypoxia/reperfusion^25^, which well explains the changes of nuclear membrane in this study.

Inhibitors of ferroptosis, apoptosis, autophagy, and necrosis, can alleviate the cell deaths of trophoblast under hypoxic conditions^19^. Kajiwara et al.^26^ have found that human trophoblast cells are sensitive to the ferroptosis induced by the depletion or inhibition of GPX4 or lipase PLA2G6. This process is characterized by giant bubbles and vesicles in the plasma membrane; these observations are characteristic markers of ferroptosis. Beharier et al.^16^ found that the mortality rate of the trophoblast cell line PHT and BeWo cells increased gradually under the action of different concentrations of the ferroptosis inducer RSL3. Furthermore, injury could only be alleviated by the administration of a ferroptosis inhibitor (Fer-1) rather than an inhibitor of apoptosis and necrosis. These results are consistent with those derived from present research and suggests that trophoblast ferroptosis occurs during the first four stages of PE.

The up-regulation of DJ-1 plays an important role in cell survival during the process of chronic OS. On the one hand, DJ-1 stimulates and maintains the normal production of ATP by directly increasing molecular chaperone activity and inhibiting p53 translocation to the mitochondria. On the other hand, DJ-1 mediates the downstream signaling pathway such as Nrf2/GPX4 to stimulate the production of superoxide dismutase (SOD) and GSH. The last three amino acids at the C-terminal of DJ-1 (Δ C3) play a key role in maintaining its dimer structure. The hydrophobic L187 residue can also alleviate the ferroptosis effect of erastin on DJ-1−/− mouse embryonic fibroblasts^27^. In a previous study, we found that the expression levels of DJ-1 mRNA and protein in the serum and placenta of patients with PE were significantly increased^28^. We also found that the up-regulated levels of Nrf2/GPX4 in the placenta of patients with severe PE enhanced the invasive ability of trophoblasts^29^. DJ-1 and the Nrf2/GPX4 signaling pathway plays a protective role in the process of ferroptosis. De Miguel C et al.^30^ found that oxidative stress hypertension occurred in DJ-1−/− mice when the expression of DJ-1 was inhibited. This was accompanied by an increased renal MDA concentration and increased expression of the oxidative stress marker heat shock protein 60 mRNA. Yao et al.^31^ constructed a rat model of sepsis-associated encephalopathy (SAE) and found that the levels of MDA and Fe^2+^ in the hippocampus of SAE rats were significantly higher than controls, while the expression levels of Nrf2 and GPX4 proteins were significantly decreased. The activation of the Nrf2/GPX4 signaling pathway by deferoxamine preconditioning could significantly reduce the levels of ferroptosis in hippocampal neurons and improve cognitive impairment. These previous research results are consistent with the conclusions of our current experiments. In the present study, we found that trophoblasts are sensitive to ferroptosis. We also confirmed that high expression levels of DJ-1 can inhibit ferroptosis in trophoblast cells via the Nrf2/GPX4 signaling pathway; we draw a conclusion that DJ-1 plays a protective role in the pathogenesis of PE.

### Advantages and disadvantages of the current research

There are several advantages to the present research. First, we verified that ferroptosis was observed in the placentas of patients with PE, that trophoblast cell lines were highly sensitive to ferroptosis, and that DJ-1 played a protective role in the pathogenesis of PE by regulating the expression levels of the downstream Nrf2/GPX4 signaling pathway. Secondly, we investigated and demonstrated the process of ferroptosis in placenta from patients with PE and identified the roles of ferroptosis, DJ-1, and the Nrf2/GPX4 signaling pathway, in trophoblast cells. The major innovative aspect of our research was identifying the relative roles of DJ-1, the Nrf2/GPX4 signaling pathway, and ferroptosis, in the pathogenesis of PE at the level of the placenta.

Similarly, there are many areas that need to be improved in our experiment. For example, in order to better illustrate the role of trophoblast ferroptosis in the pathogenesis of PE, it is necessary to measure the levels of Fe^2+^ and ferritin in placental tissue. In addition, in order to better demonstrate that DJ-1 can mediate ferroptosis in trophoblast can play a proactive role in the pathogenesis of PE through Nrf2/GPX4 signal pathway, future research should involve the comprehensive analysis of animal models of PE.

## Conclusion

In the present research, we identified that ferroptosis is involved in the pathogenesis of PE and that DJ-1 can mediate ferroptosis in trophoblast cells and play a protective role in the pathogenesis of PE by regulating the Nrf2/GPX4 signaling pathway.

## Supplementary Information

Supplementary Information.

## Data Availability

The datasets used and analyzed during the current study are available from the corresponding author on reasonable request.
