# Supplementary figures and images for: DJ-1 upregulates the Nrf2/GPX4 signal pathway to inhibit trophoblast ferroptosis in the pathogenesis of preeclampsia
Source: Sci Rep. 2022 Feb 21;12:2934. doi: 10.1038/s41598-022-07065-y (PMC8861185; doi:10.1038/s41598-022-07065-y)

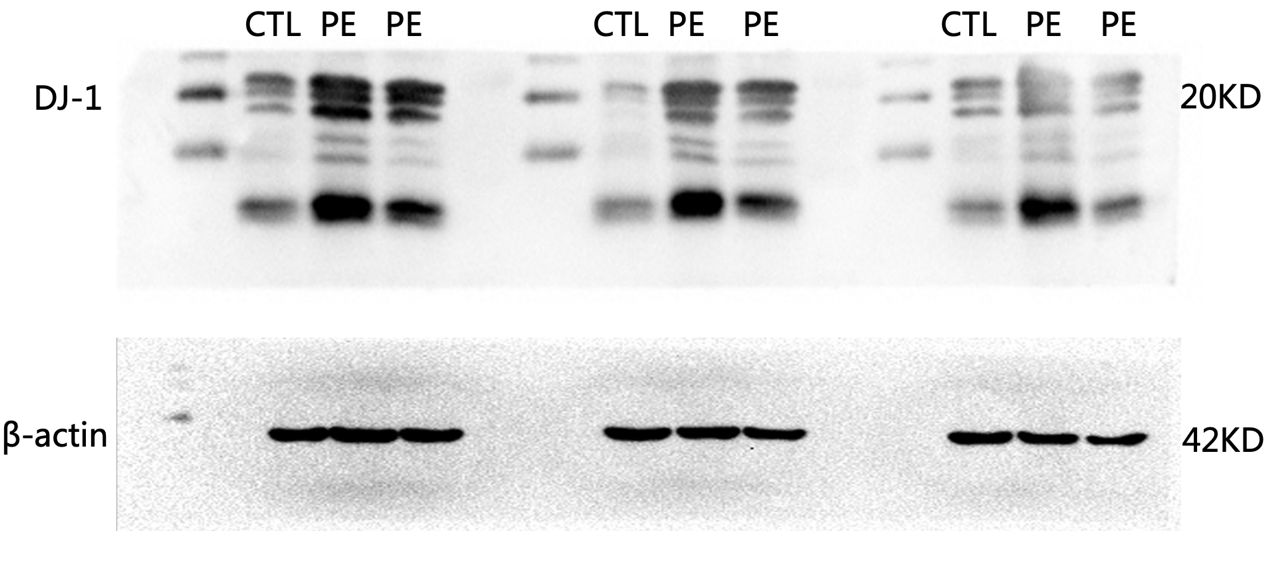


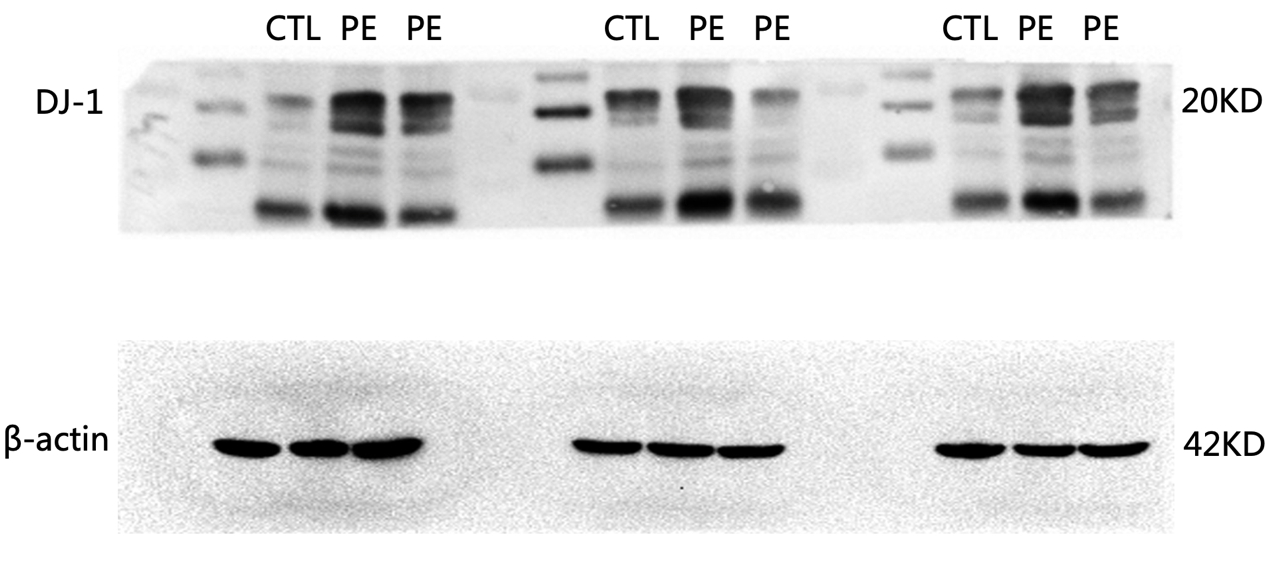


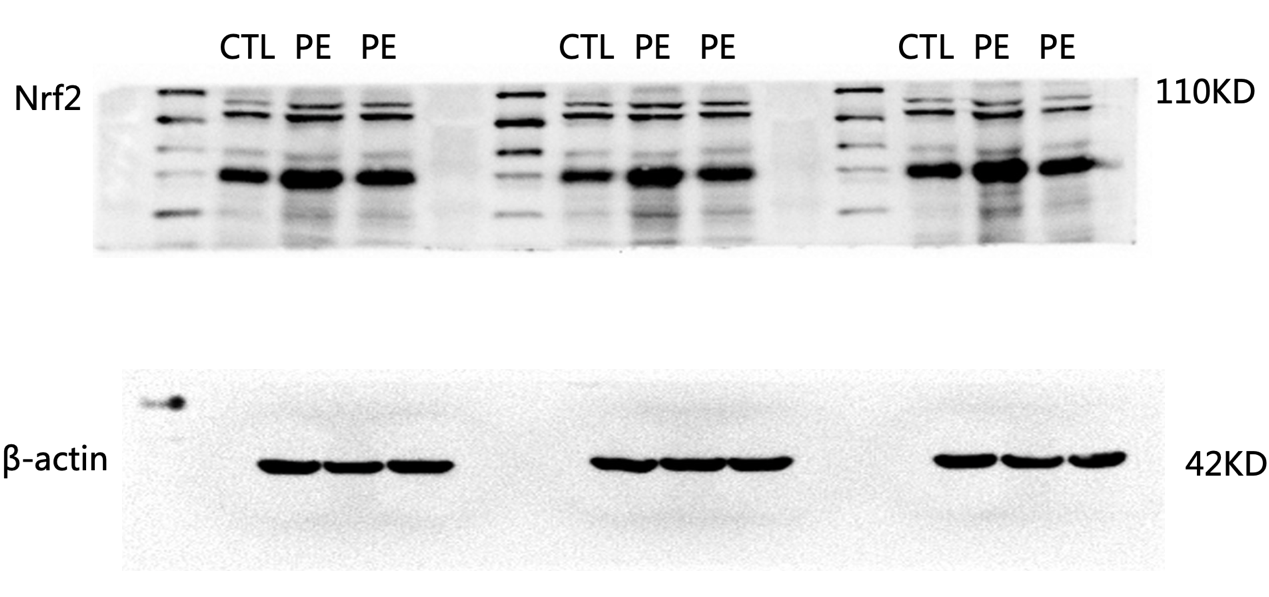


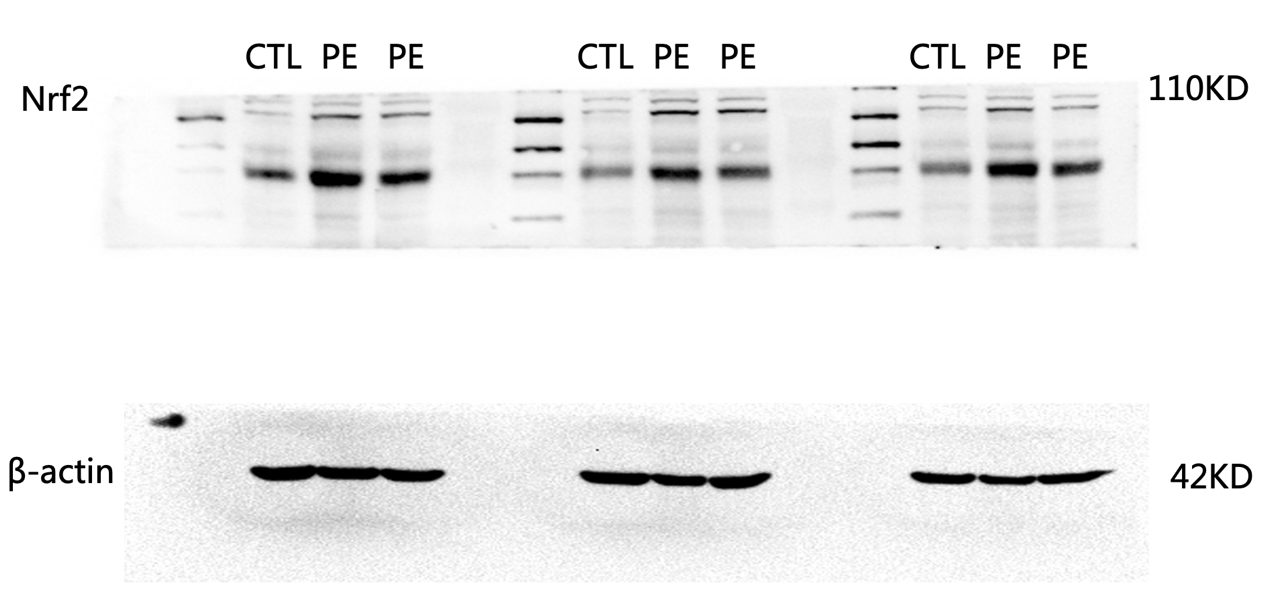


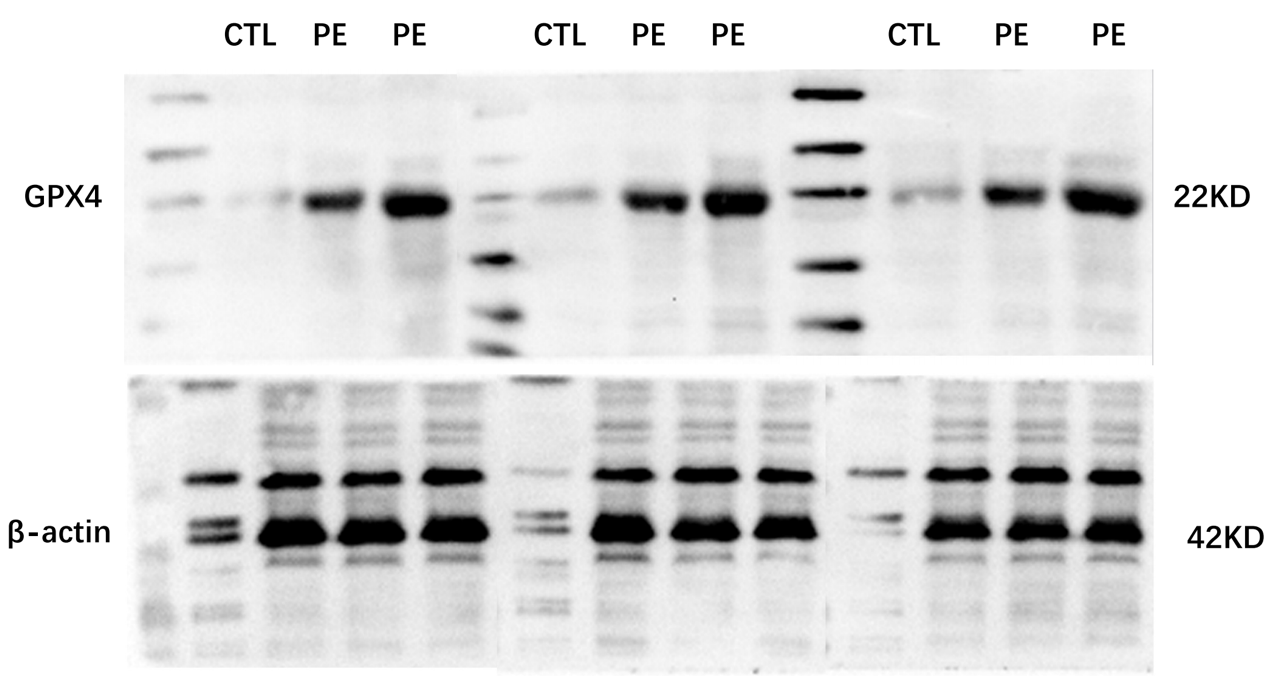


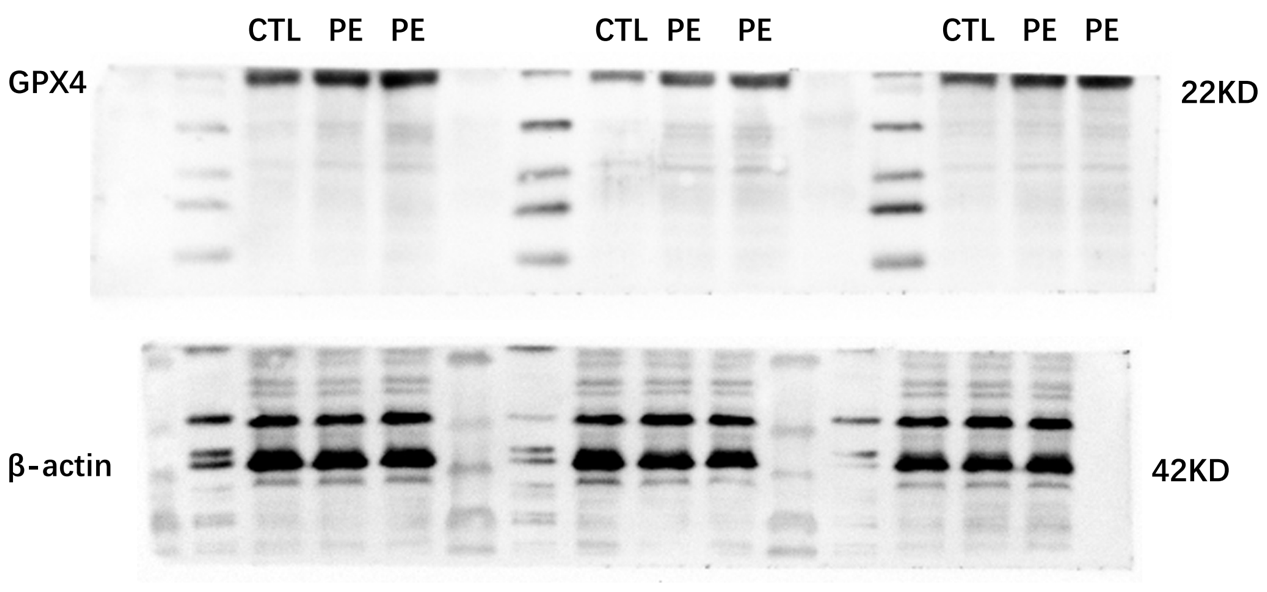

Supplement: Supplementary file 1 — Supplementary Information. [file 41598_2022_7065_MOESM1_ESM.docx]
